# iPSC-Derived Gaucher Macrophages Display Growth Impairment and Activation of Inflammation-Related Cell Death

**DOI:** 10.3390/cells10112822

**Published:** 2021-10-21

**Authors:** Daria Messelodi, Salvatore Nicola Bertuccio, Valentina Indio, Silvia Strocchi, Alberto Taddia, Salvatore Serravalle, Jessica Bandini, Annalisa Astolfi, Andrea Pession

**Affiliations:** 1 Department of Medical and Surgical Sciences, University of Bologna, 40138 Bologna, Italy; daria.messelodi2@unibo.it (D.M.); salvatore.bertuccio2@unibo.it (S.N.B.); alberto.taddia3@unibo.it (A.T.); 2 Alma Mater Institute on Healthy Planet, University of Bologna, 40138 Bologna, Italy; valentina.indio2@unibo.it; 3 Laboratory of Translational Research, Ospedale Santa Maria Nuova–IRCCS, 42123 Reggio Emilia, Italy; silvia.strocchi@ausl.re.it; 4 Division of Pediatrics, IRCCS Azienda Ospedaliero-Universitaria di Bologna, 40138 Bologna, Italy; salvatoreserravalle@libero.it (S.S.); jeba89@alice.it (J.B.); andrea.pession@unibo.it (A.P.); 5 Department of Translational Medicine, University of Ferrara, 44121 Ferrara, Italy

**Keywords:** Gaucher disease, iPSC, macrophages, inflammation, necroptosis

## Abstract

Gaucher disease is a lysosomal storage disorder characterized by β-glucosidase enzyme deficiency and substrate accumulation, especially in cells of the reticuloendothelial system. Typical features of the disease are the unrestrained activation of inflammatory mechanisms, whose molecular pathways are still unclear. To investigate biological mechanisms underlying the macrophage activation in GD, we derived iPSCs from a healthy donor and a GD patient line and differentiated them into hematopoietic progenitors. While GD iPSCs are able to efficiently give rise to CD33+/CD45+ myeloid progenitors, the maturation towards the CD14+/CD163+ monocyte/macrophages fate resulted enhanced in the GD lines, that in addition displayed a decreased growth potential compared to control cells either in semisolid or in liquid culture. The GD lines growth impairment was associated with a significant upregulation of RIPK3 and MLKL, two key effectors of necroptosis, the inflammation related cell death pathway. The activation of necroptosis, which has already been linked to neuronopathic GD, may play a role in the disease proinflammatory condition and in the identified cell growth defects. Understanding the GD macrophage role in the alteration of mechanisms linked to cellular metabolism imbalance, cell death and inflammation are crucial in identifying new ways to approach the disease.

## 1. Introduction

The understanding of pathogenetic mechanisms related to single gene alterations is sometimes more complex than expected. Monogenic diseases can have a wide spectrum of alterations being the phenotypic manifestations of different molecular mechanisms involving many cell types and their interactions. This is the case of many lysosomal storage disorders, including Gaucher disease (GD).

GD is characterized by β-glucosidase (GCase) enzyme deficiency, due to mutations in the *GBA1* gene. GCase catalyzes the hydrolysis of glucosylceramide (GluCer), playing a crucial role in the degradation of complex lipids and in the cell membrane turnover [1,2]. Mutations into *GBA1* lead to GCase structure modifications that have important effects on the enzyme activity [3]. GCase impairment causes the accumulation of different substrates including GluCer, ceramide and glucosylsphingosine, primarily in the reticulo-endothelial system cells, like macrophages, as they play a central role in the degradation of cells containing high amounts of glycosphingolipids like erythrocytes and leukocytes [4]. The substrate storage occurs primarily in the lysosomes, and it spreads throughout the cell cytoplasm, which results in them being filled with fibrillar material [5,6]. Lipid-laden Gaucher macrophages switch to an enlarged shape and start to produce a peculiar signature of inflammatory genes, including the chitin degrading enzyme, chitotriosidase, the transmembrane glycoprotein NMB (GPNMB) and scavenger receptors [7]. Because of their ability to massively infiltrate spleen, liver and bone marrow, they are considered to be the main cause of the disease’s symptomatology [8].

Even if a macrophage-centric view is not enough to explain the complex clinical picture of GD, which includes also predisposition to malignancy, autoimmune disease, Parkinson disease and osteoporosis [9], the macrophage is certainly one of the main players in the disease.

To better describe the GD macrophage features and to study the mechanisms trigging the inflammation response in the hematologic compartment, we have differentiated iPSC derived from a GD-patient and a healthy donor treated with the GCase inhibitor conduritol B epoxide (CBE) towards the monocyte/macrophage fate.

iPSCs have already proved to be a valuable tool for studying specific cell populations in GD, reproducing the primary disease phenotypes and representing a suitable model to investigate the consequences of GCase deficiency and the side effects of substrates accumulation in the cell lysosomes and cytoplasm [10,11]. We show that iPSC-derived monocyte/macrophages, lacking a proper GCase enzyme activity, have an altered growth potential, combined with the activation of the necroptosis pathway, which has already been described as one of the cell death mechanisms playing a role in GD neuroinflammation condition [12,13]. 

## 2. Materials and Methods

### 2.1. iPSC Lines Generation and Characterization

iPSCs were generated from the peripheral blood mononuclear cells (PBMCs) of a GD type 1 patient (GD-1), carrying a compound heterozygous condition for the N370S and L444P mutations in the *GBA1* gene and a healthy donor (CTRL). After blood collection, mononuclear cells were isolated through Ficoll (Lymphoprep) gradient centrifugation, collected and counted. Sendai vectors encoding for the reprogramming factors: Klf4, cMyc, Sox2 and Oct4 from the CytoTune™-iPS 2.0 Sendai Reprogramming Kit (ThermoFisher, Waltham, MA, USA) was used to reprogram the cells according to the manufacturers’ instructions. After the isolation of the first iPSC colonies, they were live-stained with TRA 1–60 pluripotency antibody (ThermoFisher) and amplified in vitronectin-coated plates. Cell karyotype was checked before and after the reprogramming process through the G-banding technique. Single iPSCs were seeded in ultra-low attachment plates (STEMCELL Technologies, Vancouver, BC, Canada) in Essential 6 medium (ThermoFisher) to evaluate their ability to give rise to embryoid bodies (EB) structures. Primer for the three germinal layers genes was used to check through quantitative Real Time PCR (qRT-PCR) their expression levels from RNA extracted from EB structure after 14 days in culture (Table S1). 

The study protocol was approved by the Ethical Committee of S.Orsola-Malpighi Hospital of Bologna (code84/2019/Sper/AOUBo). Written informed consent was obtained from all study participants or their legal guardians.

### 2.2. iPSC Culture Maintenance

iPSCs were cultivated in Essential 8 medium (ThermoFisher) on vitronectin-coated (ThermoFisher) 6-well plates (Falcon, Corning, NY, USA). For standard passaging, after DPBS wash, cells were incubated for 2 min in DPBS/0.5 mM EDTA, detached in 1 mL of fresh medium and transferred in a new vitronectin-coated plate. For procedures requiring the single cell condition, cells were detached in 1 mL of Accutase (ThermoFisher) after a 5 min incubation at 37 °C. Cells were then centrifuged for 5 min at 300× *g* and resuspended in the final medium supplemented with ROCK inhibitor Y27632 10 µM (Miltenyi, Bergisch Gladbach, Germany). iPSCs were cryopreserved in KnockOut Serum (ThermoFisher) with 10% DMSO. In addition, routinely tested for mycoplasma contamination. 

Healthy donor-derived iPSC line was treated with the GCase inhibitor CBE (Sigma-Aldrich, St. Louis, MO, USA) at 250 µM concentration to induce a GD-like condition. The treatment duration depended on the experimental protocol.

### 2.3. Monocyte–Macrophage Differentiation

iPSCs were differentiated towards the monocyte/macrophage fate using a 2D co-culture system on matrix [14]. Briefly, 5–6 colonies of each iPSC line were picked and put into Geltrex (Thermofisher)-coated wells in Essential 8 medium. The day after the medium was replaced with StemPro34 medium (Thermofisher) enriched with 1× penicillin/streptomycin (Gibco), 2 mM glutamine (Gibco), 50 µg/mL ascorbic acid (Sigma-Aldrich, St. Louis, MO, USA), 15 mM monothioglycerol (Sigma-Aldrich) and the cytokines required in the day 0 of the differentiation (Table S2). The medium was replaced every two days until day 18 with a cytokine cocktail specific for every time point of the differentiation, as shown in Table S2. At day 7, hematopoietic precursors started to emerge from the underlying stromal cells. Hematopoietic differentiation of iPSC was then assessed at day 13, 15 and 18 of the differentiation through flow cytometry analyses, checking the positivity for the hematopoietic precursors’ markers (CD34, CD45), myeloid precursor (CD33) and monocyte/macrophage lineage markers (CD11b, CD14 and CD163). All samples were analyzed through the FACSCantoII flow cytometer, and the data analyzed with the FCS Express software (De Novo Software, Pasadena, CA, USA). All the antibodies were purchased from BD and all the cytokines from Peprotech (Rocky Hill, NJ, USA).

### 2.4. iPSC-Derived Monocyte/Macrophages Growth Evaluation

Hematopoietic cells (emerged at day 15 of the differentiation protocol) were seeded both in methylcellulose (H4230, STEMCELL Technologies—50,000 cells per condition in a 6-well plate) and in the basal differentiation medium (100,000 cells/well in a 12-well plate) enriched with the proper cytokine cocktail. The number of generated colonies was evaluated every six days, and cells were counted and replated in the same condition for three passages (p1, p2, p3). For liquid culture the cell number was evaluated every three days. 

Cell Proliferation Reagent WST-1 (Sigma-Aldrich) was used to measure cell growth and proliferation 72 h after seeding 10,000 cells per well in a 96-well plate.

### 2.5. GCase Enzyme Activity

GCase enzyme activity was measured through the fluorescent substrate 4-methylumbeliferyl-N-acetyl-β-glucosamine (Sigma-Aldrich M-3633) method. The test was performed on protein lysate, extracted through sonication in PBS + Triton X-100 0.01%. 1 mg protein was incubated with 100 μL of substrate and water or CBE as negative control. After 1 h at 37°, the fluorescence level was measured using the Spark multiplate reader (Tecan, Männedorf, Switzerland). The enzyme activity was expressed as nmol/mg/h.

### 2.6. RNA Extraction and RT-PCR

Total RNA was extracted by RNeasy spin column method (Qiagen, Hilden, Germany) and 500 µg were reverse transcribed to cDNA with the Transcriptor first strand cDNA synthesis kit (Roche Diagnostics, Basel, Switzerland). Luna Universal qPCR Master Mix (NEB, Ipswich, MA, USA) was used for Quantitative Real-Time PCR (qRT-PCR) on the LightCycler 480 instrument (Roche Diagnostics) with specific primers for target genes (Table S3). DDCt method was used to quantify gene expression levels normalized on the housekeeping genes (GAPDH, ATPS and YWHAZ). 

### 2.7. Western Blot

Protein lysates were obtained from cell pellets after incubation with RIPA buffer and quantified with the Pierce BCA protein assay kit (LifeTechnologies, Carlsbad, CA, USA). A total of 30 ug of protein was loaded on a 12% gel for Western blot analysis. Blots were incubated with primary antibodies (RIPK3, pRIPK3—Abcam and MLKL, pMLKL and β actin—Cell Signalling Technologies) overnight at 4 °C on a shaker platform and were then probed with anti-mouse and anti-rabbit (Cell Signalling Technologies, Danvers, MA, USA) secondary antibodies (all 1:10,000) for 1 h at room temperature. Pictures were acquired after Clarity (BioRad, Hercules, CA, USA) incubation with ChemiDoc XRS (BioRad). Densitometry analyses on the immunoblots were performed by ImageLab software (BioRad).

## 3. Results

### 3.1. GD-iPSC Efficiently Differentiate towards the Macrophage Fate

**Figure 1:**
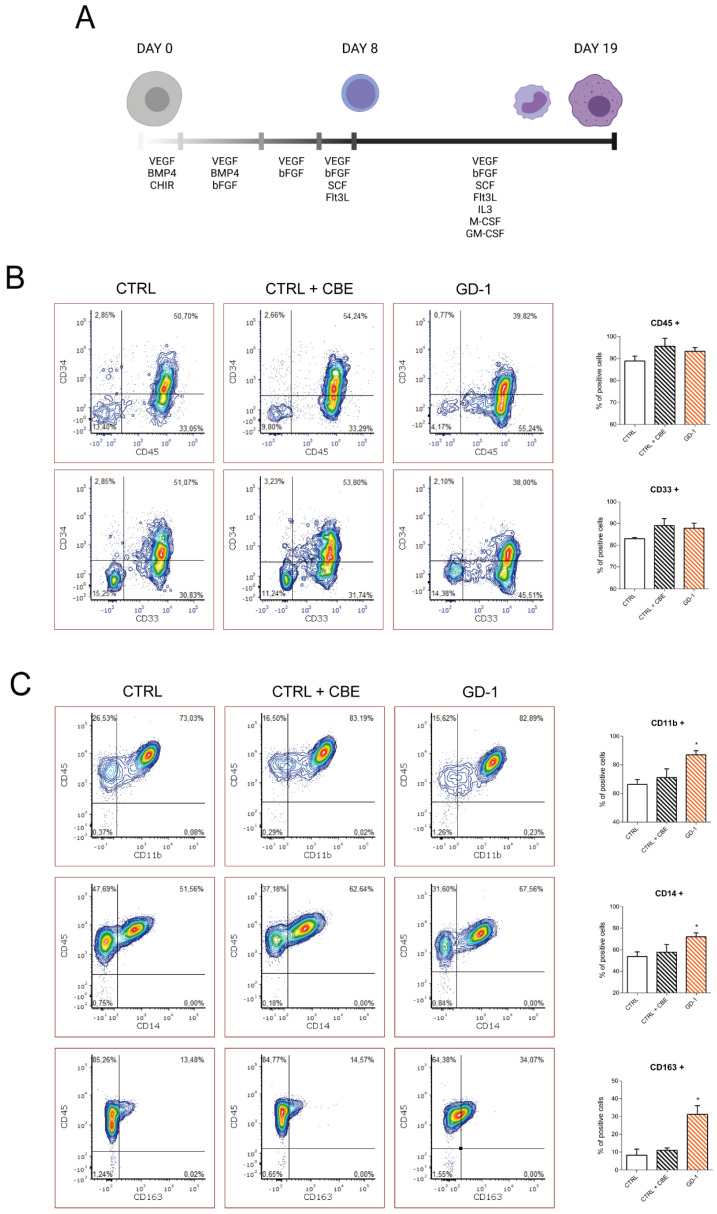
GD-iPSC efficiently differentiate into CD11b, CD14, CD163 monocyte/macrophages. (**A**) Schematic representation of the differentiation protocol used to obtain mature macrophages starting from iPSC going through the hematopoietic precursor stage. The main cytokines added to the growth medium are reported. (Created with BioRender.com) (**B**) Flow cytometry evaluation of hematopoietic progenitors at day 9 of differentiation protocol stained with CD34 and the myeloid precursor markers CD33 and CD45. The graph shows mean values and SEM of the percentage of positive cells. *n* = 3. (**C**) Mature iPSC-derived monocyte/macrophage cells positivity for CD11b, CD14 and CD163 markers at day 19 of the differentiation protocol. The graph shows mean values and SEM of the percentage of positive cells. *n* = 3. * *p* < 0.05.

After the generation and characterization of CTRL and GD type 1 patient iPSC (Figure S1A–C), they were amplified and subsequently differentiated into hematopoietic precursors and monocyte/macrophage cells through a 2D coculture system on matrix (Figure 1A). From day 9 of the differentiation protocol, CTRL cells were treated with CBE 250 µM to inhibit GCase enzyme and obtain a GD-like condition (Figure S1D). The supernatant containing the hematopoietic precursors cells emerged from the underlying stroma was collected and lineage specific markers were tested through flow cytometry starting from day 9 of differentiation. CTRL cells, either untreated or treated with CBE, and GD-1 lines were all able to give rise to CD34+/CD45+ hematopoietic progenitor cells, as well as myeloid precursor CD45+/CD33+ (Figure 1B). After 10 other days in culture under specific cytokine stimuli, cells acquired the mature monocyte lineage markers CD11b, CD14 and partially also the mature macrophage marker CD163. Interestingly, GD-1 line expressed significantly higher levels of all the analyzed markers if compared with the CTRL. The same trend, even if not significant, emerged also analyzing cells treated with CBE (Figure 1C) suggesting that GCase deficit may enhance the myeloid differentiation efficiency, with a higher production of monocyte/macrophage cells.

### 3.2. GD-iPSC-Derived Monocyte/Macrophage Cells Display a Growth Defect

**Figure 2:**
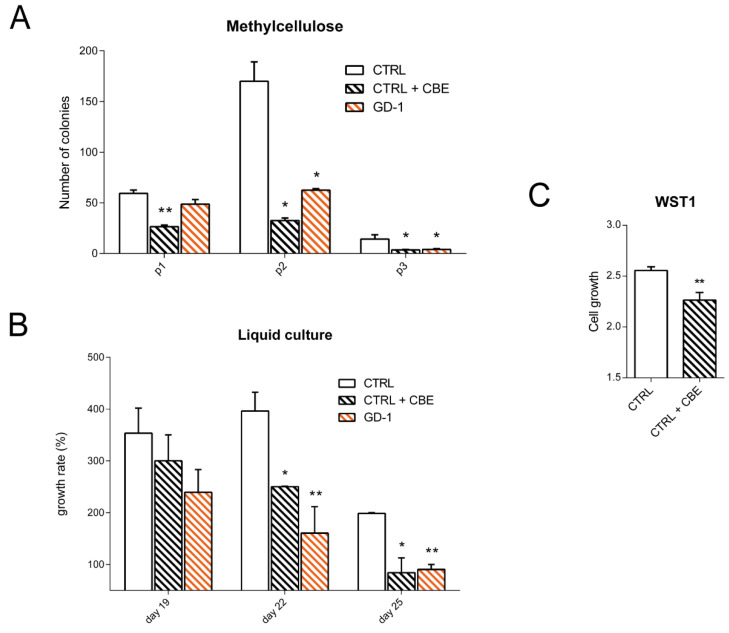
iPSC-derived monocyte/macrophage cells display a decreased cell growth rate. (**A**) Number of the colonies of CD45+ cells seeded in semi-solid culture condition represented as mean with SEM (*n* = 3 independent experiments). Cells were seeded and replated every 6 days for three times (p1, p2, p3). CBE 250 µM was added to CTRL cells at every replating. (**B**) Count of the iPSC-derived monocyte/macrophages growth rate seeded in liquid culture with VEGF 50 ng/µL, bFGF 50 ng/µL, SCF 50 ng/µL, Flt3L 5 ng/µL, IL3 25 ng/µL, M-CSF 50 ng/µL, GM-CSF 25 ng/µL. For CTRL + CBE condition, CBE 250 µM was added to the medium at every cell passage. Graphs represent mean with SEM (*n* = 3). (**C**) Measurement of cell proliferation with the WST1 reagent of CTRL and CTRL treated with CBE iPSC-derived monocyte/macrophages after 19 days of differentiation. Histograms represents mean with SEM. Statistical significance is indicated (Student’s *t*-test), * *p* < 0.05, ** *p* < 0.01.

At day 15 of the differentiation protocol cells were collected and seeded both in semisolid and liquid culture conditions enriched with hematopoietic cytokines to check for their self-renewal properties and proliferative rate. After 6 days in methylcellulose the number of emerged colonies were counted and the cells were replated in the same conditions. From the first time point, CTRL cells treated with CBE and GD-1 cells showed a lower capacity to give rise to colonies, a condition that became more evident after the first and the second replating (Figure 2A). A similar situation was evident also in the liquid culture analysis where iPSC-derived monocyte/macrophages were counted and reseeded every three days. The growth defect of GD lines compared with CTRL appeared statistically significant and seemed to be closely related to the enzymatic defect since the treatment with CBE caused a deficit similar to the patient derived cells (Figure 2B). The WST1 cell viability assay further proved the reduced cell proliferation in CBE-treated cells compared with CTRL line (Figure 2C), suggesting that the defect is linked with the lack of GCase functionality and not related to the possible gain of function of mutant enzyme.

### 3.3. Necroptosis Pathway Is Hyperactivated in GD Monocytes/Macrophages

To test whether this growth impairment is related to increased cell death signals to which cells are subjected in the disease context, we tested the activation status of necroptosis pathway along with cell differentiation.

**Figure 3:**
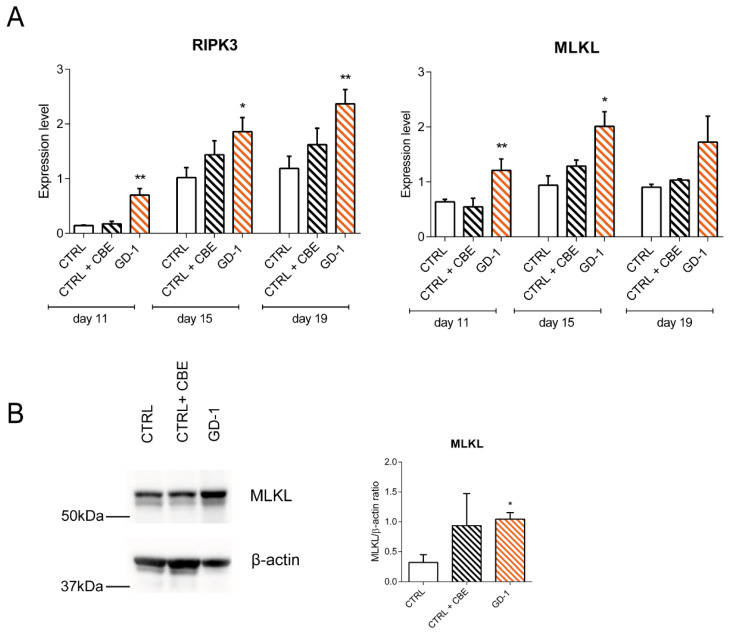
RIPK3 and MLKL are upregulated in GD iPSC-derived monocyte/macrophages. (**A**) Expression levels of RIPK3 and MLKL genes in iPSC-derived CTRL and GD lines at different time points during macrophage differentiation (day 11, 15 and 19). Expression levels are normalized with respect to three housekeeping genes. Graphs represent mean with SEM, *n* = 3. (**B**) Protein level of MLKL with the relative quantification, *n* = 2. Statistical significance is indicated (Student’s *t*-test), * *p* < 0.05, ** *p* < 0.01.

RNA was extracted from cells at different time points of the macrophage differentiation (day 11, 15 and 19) and the expression levels of the three main pathway effectors were analysed. While the mRNA levels of RIPK1 were not significantly different between the CTRL and GD cell models, the expression levels of RIPK3 and MLKL were both strongly induced in the GD-1 line compared to the CTRL, suggesting that necroptosis is highly activated when the GCase enzyme is not structurally and functionally present into the cell (Figure 3A). To further prove this assumption, RIPK3 and MLKL protein levels were tested. MLKL protein levels resulted significantly higher in GD-1 monocytes/macrophage cells, meaning that higher expression levels of the final effector of the necroptosis pathway is coupled with higher protein levels prompting the pathway activation (Figure 3B).

## 4. Discussion

The development of patient-derived iPSC had a big impact on lysosomal storage disorder research. They were proved to be able to faithfully recapitulate disease characteristics, biochemical and cellular features, giving novel opportunities to clarify the etiopathology and to test new therapeutic strategies. iPSC allowed new insights into many aspects of GD molecular and cellular pathology. Alterations in different cell mechanisms, including inflammatory response, autophagic flux, lysosomal activity, calcium signaling and α-synuclein accumulation were linked to GCase deficit and GluCer accumulation [15]. iPSC-derived macrophages were used as a platform to investigate typical GD cell features as the impaired ROS production, reduced chemotaxis [16] and production of high levels of cytokines, such as TNFα, IL-6 and IL-1β [10].

Using both healthy donor and GD type 1 patient-derived iPSCs, we were able to characterize cells’ differentiation potentials, confirming that GCase deficiency does not affect hematopoietic progenitors’ formation, but that GD lines give rise to a bigger population expressing the monocyte/macrophage lineage markers CD11b, CD14 and CD163. It has already been reported that iPSC-derived GD hematopoietic progenitors demonstrate a marked lineage commitment with increased myeloid differentiation and decreased erythroid maturation, giving rise to adherent, macrophage-like cells, indicators of abnormal myelopoiesis [17]. The increased production of monocyte/macrophages cells could be the result of an increased need for scavenger functions in GD cells due to the substrates accumulation. Different stimuli can indeed affect cell differentiation and maturation that, due to GCase impairment, are anyway not able to give rise to cells with a functional phagocytic activity.

Moreover, the evaluation of colony formation potential and the proliferation capacity of our iPSC-derived monocyte/macrophages suggested that GD mature cells do not present a normal growth rate, implying an alteration in the proliferation/death induction balance in these cells. 

As the triggering of inflammatory mechanisms and the release of cytokines are common features of GD pathophysiology [18,19], we focused our attention on inflammation-related cell death pathways. Especially at the neuronal level, it has been reported that neuroinflammation is accompanied by the activation of inflammation mediated cell death mechanisms, as necroptosis [12,13]. Considering that the activated macrophages foster a chronic stimulation of the immune system [20] with the release of pro-inflammatory cytokines [21,22] also from cells surrounding the Gaucher cells (MCP-1and IL-1β) [7], we hypothesized that the upregulation of this pathway is present also at peripheral level in differentiated myeloid hematopoietic cells and monocyte/macrophages.

Necroptosis is a programmed and regulated necrosis process described for the first time almost ten years ago [23]. It represents a cellular response to environmental stress that can be induced by inflammation, chemical and mechanical injury or infection and the activation signal is probably mediated by the TNF- receptor system [24,25]. Necroptosis modulation has been proposed as a therapeutic strategy to face different kind of homeostasis and inflammation-related, neurodegenerative and heart diseases [26].

Interestingly, we found higher expression levels of RIPK3 and MLKL, the two downstream components of the pathway, proving that this pathway plays a role in triggering inflammation-related cell death processes in GD also in the hematopoietic compartment. Although these data require further validation, considering that were carried out only on few iPSC lines, the role of the kinase RIP3 is particularly interesting since it acts not only as the critical regulator of the necroptosis pathway but also as an interactor in cell death independent inflammation processes. It does indeed play a role in the activation of the NLRP3 inflammasome, a signaling complex mediating the processing of proinflammatory molecules, such as IL-1bβ and IL-18, which are precursors in myeloid cells [27].

A better understanding of the inflammation-related features of GD macrophages is crucial to face different aspects of the disease that still remain unsolved. Enzyme replacement and substrate reduction therapies are indeed able to reverse the majority of symptoms and prevent many complications in patients without neurological involvement, but a delay in the diagnosis, that is not a rare event given GD relatively nonspecific symptoms, can lead to irreversible cell and tissue damages [28,29]. 

In conclusion, GD iPSC-derived macrophages represent a valuable tool to fill the gap in the knowledge regarding the causes and consequences of the GD pro-inflammatory condition. The enhanced maturation of hematopoietic precursors towards the monocyte/macrophage fate and the impairment of cell proliferation in GD iPSC-derived lines observed in this study represent the cellular manifestation of underlying molecular alterations, providing us with clues as to new targetable pathways, including the modulation of RIPK3/MLKL axis and its interactors which can provide interesting approaches to the disease.

## Data Availability

The data presented in this study are available on request from the corresponding author. The data are not publicly available due to privacy issues.

## Supplementary Materials

The following are available online at https://www.mdpi.com/article/10.3390/cells10112822/s1, Figure S1, Table S1: Primer list for embryoid bodies evaluation, Table S2: List of cytokines for the monocyte/macrophage differentiation, Table S3: List of qRT-PCR primers.
