# Supplementary material for: iPSC-Derived Gaucher Macrophages Display Growth Impairment and Activation of Inflammation-Related Cell Death
Source: Cells. 2021 Oct 21;10(11):2822. doi: 10.3390/cells10112822 (PMC8616237; doi:10.3390/cells10112822)
Supplement: Supplementary file 1 [file cells-10-02822-s001.zip › cells-1408947-supplementary.pdf]

Table S1. Primer list for embryoid bodies evaluation. Sequence of the primers specific for the three germinal layer and pluripotency markers used to characterize the ability of newly generated iPSC to spontaneously give rise to cells belonging to the endoderm, neuroectoderm and mesoderm layer.

**Gene**	**Primer F**	**Primer R**
NANOG	5' - CCATCCTTGCAAATGTCTTCTG - 3'	5' - CTTTGGGACTGGTGGAAGAATC - 3'
OCT4	5' - GCAGCAGATCAGCCACATC - 3'	5' - CTTGATCGCTTGCCCTTCT - 3'
AFP1	5' - CAAAATGCGTTTCTCGTTGCT - 3'	R: 5' - GCTGCCATTTTTCTGGTGATG - 3'
FOXA2	5' - CCACCTGAAGCCGGAACA - 3'	5' - TGCTCCGAGGACATGAGGTT - 3'
GATA4	5' - GCTATGCGTCTCCCGTCAG - 3'	5' - GTGACTGTCGGCCAAGACC - 3'
HAND1	5' - AACTCAAGAAGGCGGATGG - 3'	5' - AGGGCAGGAGGAAAACCTT - 3'
MIXL1	5' - GGTACCCCGACATCCACTTG - 3'	5' - TAGCCAAAGGTTGGAAGGATTTC - 3'
NEUROD1	5' - GAGCACGAGGCAGACAAGAAG - 3'	5' - CCCCCGTTCCTCAGTGAGT - 3'
PAX6	5' - CCGCCCTGGTTGGTATCC - 3'	5' - TTGGTATTCTCTCCCCCTCCTT - 3'
SOX1	5' - ATGAAGGAGCACCCGGATTA - 3'	5' - CTTCTTGAGCAGCGTCTTGGT - 3'

Table S2. List of cytokines for the monocyte/macrophage differentiation. The different cytokines have been added to the enriched StemPro34 medium for the differentiation of the iPSC towards the mature fate at different time points of the protocol.

**Day**	BMP4	CHIR	VEGF	bFGF	SCF	Flt3L	IL3	M-CSF	GM-CSF
0	5ng/mL	930ng/mL	50ng/mL						
1	5ng/mL		50ng/mL	20ng/mL					
4			15ng/mL	5ng/mL					
6			50ng/mL	50ng/mL	50ng/mL	5ng/mL			
7			50ng/mL	50ng/mL	50ng/mL	5ng/mL	25ng/mL	50ng/mL	
8			50ng/mL	50ng/mL	50ng/mL	5ng/mL	25ng/mL	50ng/mL	25ng/mL
11			50ng/mL	50ng/mL	50ng/mL	5ng/mL	25ng/mL	50ng/mL	25ng/mL
13			50ng/mL	50ng/mL	50ng/mL	5ng/mL	25ng/mL	50ng/mL	25ng/mL
15			50ng/mL	50ng/mL	50ng/mL	5ng/mL	25ng/mL	50ng/mL	25ng/mL
18			50ng/mL	50ng/mL	50ng/mL	5ng/mL	25ng/mL	50ng/mL	25ng/mL

Table S3. List of qRT-PCR primers. Sequence of the primers used to evaluate the expression levels of necroptosis pathways effectors and the relative housekeeping genes.

Gene	Pathway	Primers
RIP1	Necroptosis	F: 5' - GCAGTACCTTCAAGCCGGTC - 3'
		R: 5' - GCAAACCAGGACTCCTCCAC - 3'
RIP3		F: 5' - ACCCCGGAGCCAAATCC - 3'
		R: 5' - AGCCCCTCCCCTTGCC - 3'
MLKL		F: 5' - TCACACTTGGCAAGCGCATGGT - 3'
		R: 5' - GTAGCCTTGAGTTACCAGGAAGT - 3'
ATPS	Housekeeping	F: 5' - GTCTTCACAGTTCATATGGGGA - 3'
		R: 5' - ATGGGTCCCACCATATAGAAGG - 3'
GAPDH		F: 5' - AGGGGAGATTCAGTCTGG - 3'
		R: 5' - CGACCACTTTGTCAAGCT - 3'
RLP0		F: 5' - CCTCATATCCGGGGGAATGTG - 3'
		R: 5' - GCAGCACTGGCACCTTATTG - 3'

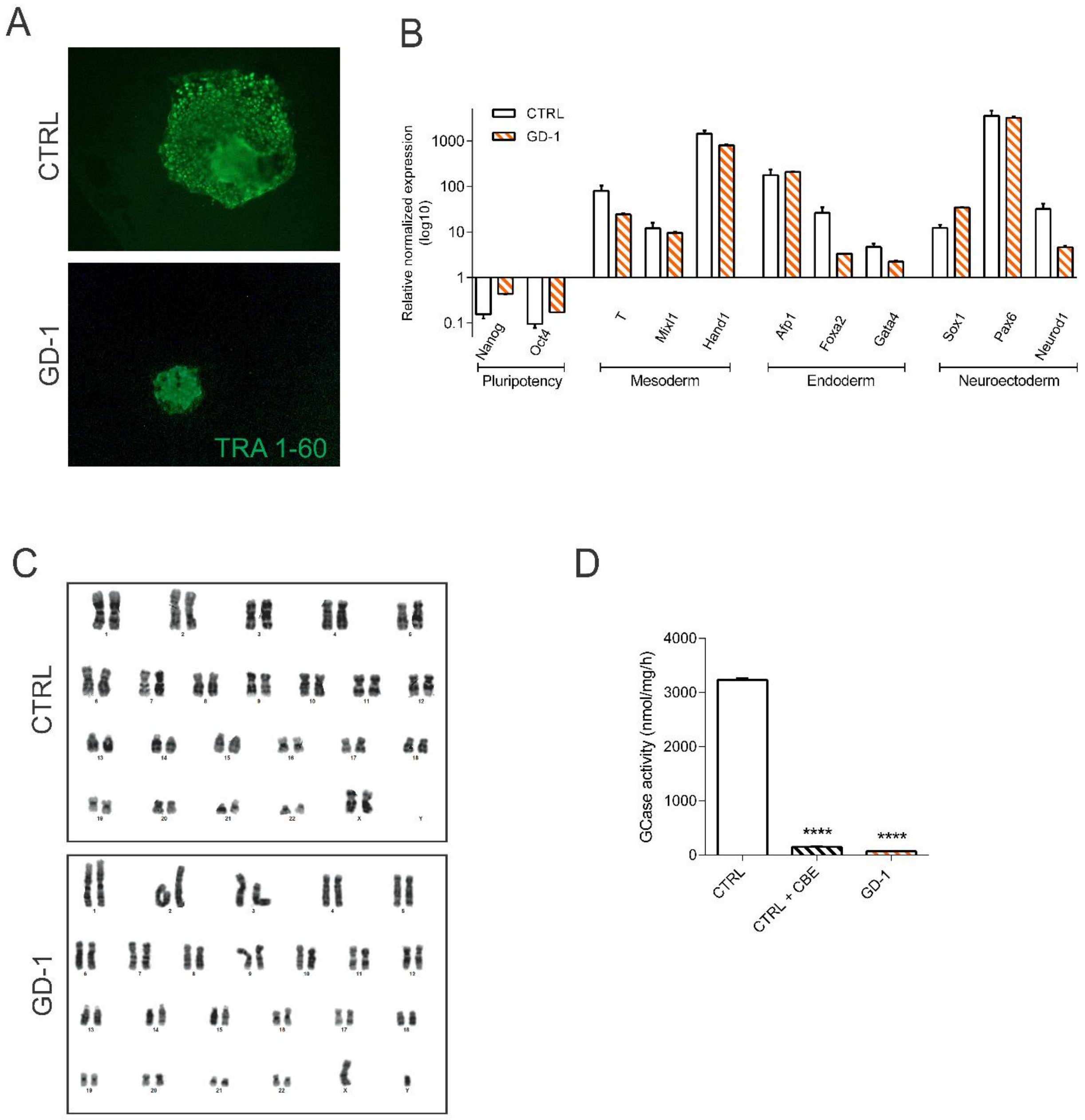

Figure S1. **A.** iPSC colony derived from the reprogramming of blood mononuclear cells isolated from a healthy donor (CTRL) and the GD type 1 patient (GD-1) stained with the TRA1-60 antibody to evaluate the pluripotency of the cells. **B.** qRT-PCR measurement of the embryonic layer markers expression after generation of embryoid bodies (EB) in both healthy donor and GD iPSC. iPSC in low attachment culture condition spontaneously generate EB expressing markers from all the three germ layers and have strongly reduced level of the pluripotency markers (Nanog and Oct4). All data are normalized on the genes' expression level in iPSC. **C.** Karyotype evaluation of the two lines after the reprogramming process performed with the G-banding technique. **D.** GCase activity measurement highlighting the strong reduction of the enzyme activity in the CBE-treated and patient-derived line. Statistical significance is indicated as p-value (Student's t-test), **** p<0.0001.
